# “I Felt Powerful and Confident”: Women’s Use of What They Learned in Feminist Sexual Assault Resistance Education

**DOI:** 10.1177/03616843211043948

**Published:** 2021-10-08

**Authors:** Sara E. Crann, Charlene Y. Senn, H. Lorraine Radtke, Karen L. Hobden

**Affiliations:** 1 Department of Psychology, 8637 University of Windsor, Windsor, ON, Canada; 2 Women’s and Gender Studies, 8637 University of Windsor, Windsor, ON, Canada; 3 Department of Psychology, 2129 University of Calgary, Calgary, AB, Canada

**Keywords:** sexual assault, rape, risk reduction, sexual assault resistance, empowerment self-defense, intervention, university women

## Abstract

Research on women’s response and resistance to sexual assault risk has informed the development of interventions to improve women’s ability to effectively resist sexual assault. However, little is known about how women anticipate, navigate, and respond to risk following participation in sexual assault risk reduction/resistance education programs. In this study, we examined the information and skills used by university women who had recently completed the effective Enhanced Assess, Acknowledge, Act (EAAA) sexual assault resistance program. We analyzed responses from 445 women using descriptive statistics and content and thematic analysis. Just under half (42%) of women used at least one EAAA strategy in the following 2 years. Most women reported that their efforts were successful in stopping an attack. Women’s responses included strategies both to preempt sexual assault threat (e.g., avoiding men who display danger cues, communicating assertively about wanted and unwanted sex) and to interrupt or avoid an imminent threat (e.g., yelling, hitting, and kicking). Women’s use of resistance strategies worked to subvert gendered social norms and socialization. The results suggest that counter to criticisms that risk reduction/resistance programs blame women or make them responsible for stopping men’s violence, women who took EAAA typically positioned themselves as agentic and empowered in their resistance.

## Introduction

Despite reductions in the prevalence of most other major crimes, rates of sexual assault have remained consistent (Koss et al., 1987; Koss & Oros, 1982; Smith et al., 2018). Sexual assault includes a range of nonconsensual sexual experiences, from unwanted sexual contact to attempted and completed rape (Edwards et al., 2014; Gilmore et al., 2018). Prevention efforts have focused on college and university campuses because of the high rates of sexual victimization reported among young women attending those institutions. Campus prevalence rates vary across studies as a function of how sexual victimization was conceptualized and measured, research design, follow-up time period, sampling, and sample characteristics (see Fedina et al., 2018, for a review), but leading experts in the field generally agree that 20–25% of undergraduate women experience sexual assault (Koss et al., 1987; Koss & Oros, 1982; Krebs et al., 2007).

Although anyone can be a victim or perpetrator of sexual assault, research has consistently documented the gendered nature of these crimes. Women are victims in 70–92% of sexual assaults and have victimization rates approximately five times higher than men (Brennan & Taylor-Butts, 2008; Sinha, 2013). Cisgender men are perpetrators in approximately 98% of sexual assaults against cisgender women (Brennan & Taylor-Butts, 2008; Martin et al., 2020). Research also consistently demonstrates that the perpetrator is known to the victim in most (75–80%) sexual assaults (Brennan & Taylor-Butts, 2008; Sinha, 2013). Sexual assault victims experience a myriad of negative psychological, emotional, neurocognitive, physical health, relational, and social outcomes that have been well-documented (DePrince & Gagnon, 2018; Jozkowski & Sanders, 2012; Ullman & Brecklin, 2002).

The perpetrator is responsible for sexual violence; however, data from large-scale victimization surveys, police reports, rape crisis center reports, and community samples document that the use of forceful verbal (e.g., yelling) and physical (e.g., hitting and kicking) resistance strategies, as well as leaving, in response to sexual assault threat reduce the likelihood of experiencing completed rape (Tark & Kleck, 2014; see Ullman, 1997, for a review). Nevertheless, various psychological barriers (many associated with the socialization of girls and women to prioritize others’ well-being above their own to preserve relationships) and other factors (e.g., alcohol consumption) impact women’s ability to recognize sexual assault risk, particularly from male acquaintances, and subsequently, to respond in ways that undermine risk. The cognitive ecological model (Nurius & Norris, 1996) posits that women undergo a multi-stage cognitive appraisal process through which they evaluate what the situation implies for their own well-being before responding to a sexual assault threat. Consistent with this, women often do not react with immediate force to a sexual assault threat from male acquaintances, because they either do not acknowledge the situation or men’s behavior as dangerous or they encounter emotional barriers to taking action against an acquaintance (Nurius & Norris, 1996).

Sexual assault risk reduction and resistance interventions help women more accurately detect risk, overcome barriers to resistance, and effectively fight back (Gidycz, et al., 2006; Orchowski et al., 2010; Rozee & Koss, 2001; Senn et al., 2011). With increasing demands for campus-based prevention, alongside evidence that very few primary prevention programs designed for boys/men and mixed-gender groups in primary through secondary school and college, produce decreases in perpetration (see DeGue et al., 2014, for systematic review; see Gidycz et al., 2011; Salazar et al., 2014, for approaches combining social norms and bystander content that have short-term impact), effective risk reduction/resistance programs to reduce victimization are increasingly being implemented. As such, it is important for researchers, institutional administrators, and program implementers to understand how women incorporate evidence-based resistance strategies from these programs into their lives. In this study, we examined university women’s accounts of their use of the information and skills acquired from an effective sexual assault resistance program over the 2 years following completion of the program.

### Women’s Responses and Resistance to Sexual Assault Threat

Research studying women’s responses to sexual assault threat has identified a number of influential factors and informed the development and improvement of risk reduction/resistance interventions (Edwards et al., 2014). This research can be categorized into three types: (a) identifying the tactics women employ to reduce the likelihood (i.e., risk) that she will be the target of sexual assault, otherwise known as protective behavioral or precautionary strategies (PBS), (b) measuring women’s behavioral responses to either a hypothetical or real sexual assault threat, also labeled sexual assault resistance strategies, and (c) identifying the risk reduction/resistance and self-defense strategies used by women following sexual assault risk reduction/resistance education.

#### Sexual Assault Protective Behavioral/Precautionary Strategies

Forty years of research has documented various ways in which women modify their behavior to avoid sexual victimization (e.g., Riger et al., 1978; Stanko, 1987). For example, Hickman and Muehlenhard (1999) identified 29 precautionary behaviors that women employed to avoid acquaintance and stranger rape, including avoiding going on dates, being careful about clothing choices to avoid “sending mixed signals,” and avoiding outdoor behaviors such as walking alone at night. Notably, women reported engaging in higher levels of these precautionary behaviors due to fear of stranger rape than acquaintance rape, which reflected how societal rape myths guided their conduct.

Women who have been sexually assaulted employ the same or similar protective behavioral strategies in an effort to prevent future victimization. In interviews with sexual assault survivors, Ullman et al. (2018) identified a number of post-assault strategies, including day-to-day behavioral changes such as changing travel routes and jobs; self-defense strategies such as carrying a weapon, becoming more assertive with men, and not going out alone at night; and avoiding or reducing drinking and drug use. As Ullman et al. (2018) pointed out, survivors (and women generally) internalize these victim-centered precautionary strategies, reflecting a certain level of rape myth acceptance (i.e., sexual assault only happens in certain situations, by avoiding those situations you can avoid sexual assault, and women are responsible for making such changes to avoid rape). Notably, Ullman et al. (2018) acknowledged the great lengths women went to avoid revictimization, often placing extreme restrictions on their lives without the benefit of increased safety, particularly in acquaintance situations. This echoes Stanko’s (1987) critique about the negative impact of these types of precautions on the quality of women’s lives, both for survivors and for those who have never been victimized.

#### Women’s Behavioral Responses to Imminent Sexual Assault Threat

Despite the myriad of protective strategies that women employ in response to the threat of sexual assault (re)victimization, a sizable minority of men continue to use coercion and force against women to exert power and control through sexual violence (Swartout et al., 2015). Research examining women’s responses to imminent sexual assault threat using hypothetical situations to ascertain how women are likely to respond to such threats found that undergraduate women were more likely to respond with verbal and physical assertiveness (e.g., verbally refusing or enforcing boundaries and moving away from the threat) than with non-assertive tactics (e.g., complying, making excuses, crying, freezing, and bargaining; Anderson et al., 2016; Masters et al., 2006). Assertive responses including physical resistance and calling for help became more likely as the threat escalated (Anderson et al., 2016; Masters et al., 2006). Women’s intention to use assertive and forceful verbal and physical resistance in response to hypothetical sexual assault threat from an acquaintance is encouraging. In fact, Masters et al. (2006) argued that some women’s descriptions of their responses reflected women’s empowerment in their willingness to respond with physical resistance in a dating scenario. However, consistent with the emotional obstacles to risk detection and resistance (Nurius & Norris, 1996), Masters et al. (2006) noted that these descriptions often reflected traditional sexual scripts wherein coercion was constructed as part of a continuum of heterosexual sexuality with an emphasis on maintaining relationships and reducing interpersonal conflict.

There is also some contradictory evidence that suggests women are unlikely to respond forcefully to a hypothetical threat of sexual assault. Norris et al. (1996) found that sorority college women were moderately likely to use “gentle or indirect messages” such as “jokingly telling him he is coming on too strong” and had a low likelihood of being verbally assertive (e.g., “raise your voice and use stronger language”) or using physical resistance (e.g., hitting and kicking) in response to a hypothetical situation where a male acquaintance was pressuring them to have unwanted sex after consensual kissing.

While research on women’s resistance using hypothetical scenarios advances our understanding of how women might respond to sexual assault threat, the results may not generalize to real-life situations. Moreover, the mixed findings of such research suggest other research designs are needed for a comprehensive understanding of women’s sexual assault resistance. Few studies have examined women’s real-life responses to such threats, and those that have show that women do not consistently use strategies proven to be most effective. Edwards et al. (2014) used qualitative methods to examine women’s resistance strategies (as well as perpetrator tactics) in a recent sexual assault situation. Participants typically reported using resistance strategies that paralleled perpetrator tactics (e.g., responding to verbal pressure with verbal resistance), although this was not true for all women. While women employed verbal resistance such as saying “no” and “stop” where the perpetrator used verbal or physical tactics such as nagging and pleading or physically holding her down, only one-third of women who described situations where the perpetrator used physical tactics responded with physical resistance. Almost 60% of women reported using non-forceful resistance strategies in response to a perpetrator’s (forceful) physical tactics such as pleading, passively moving away, pretending to sleep, and crying, which are largely ineffective in stopping a sexual assault (e.g., Ullman, 1997, 2007).

Turchik et al. (2007) used a prospective design to examine psychological and situational factors predicting women’s actual use of resistance strategies in a real-life sexual assault. Women who reported the intention to use forceful strategies or greater confidence in using such strategies at baseline were more likely to report using assertive resistance strategies in response to a real-life sexual assault threat 2 months later. Conversely, intention to use non-forceful resistance strategies, greater feelings of self-consciousness, and knowing the perpetrator predicted use of non-forceful strategies. In a similar prospective study, Gidycz et al. (2008) found that women’s intention to use assertive resistance strategies and offender aggression (use of physical restraint) predicted use of assertive strategies in response to a sexual assault threat 9 weeks later. Thus, while research using hypothetical situations demonstrates that a sizeable proportion of women would use assertive verbal and physical resistance (and would escalate their resistance in response to increasing threat), other studies highlight potential psychological and emotional barriers to using these strategies in real life, such as feelings of self-consciousness and knowing the perpetrator.

#### Women’s Behavioral Response to Threat Following Sexual Assault Risk Reduction/Resistance Education

Although women’s responses to sexual assault threat may include both forceful and non-forceful strategies, the evidence on the relationship between resistance and victimization is clear—women who use forceful resistance strategies are less likely to experience a completed rape (Tark & Kleck, 2014; Ullman, 2007; Ullman & Knight, 1992). However, previous research shows that only 20 to 25% of women use forceful resistance in response to rape attempts (Ullman, 2007). This is not surprising considering that fewer than one in five women have taken self-defense training of any kind (Runyan et al., 2007), and most self-defense programs focus on physical or sexual stranger attacks, the least common forms of sexual assault threat (Hollander, 2016; Schorn, 2015). Responding to this research, sexual assault risk reduction interventions (purposefully reframed as resistance interventions by some scholars; Senn et al., 2018) aim to empower women to use a range of self-protective behaviors (including assertive/forceful self-defense strategies) to help women develop skills to assert their intentions and resist sexual victimization (Calhoun et al., 2012; Gidycz, et al., 2006; Orchowski et al., 2010). These interventions typically target some combination of modifiable risk factors at individual, relational, and contextual levels to reduce women’s risk and improve targeted outcomes (e.g., reduced victimization rates; Edwards & Sessarego, 2018). Modifiable risk factors in resistance interventions include individual-level factors such as attitudes (e.g., stereotyped gender roles), ability to accurately recognize sexual assault risk in situations and men’s behavior (Gidycz et al., 2006), overcoming delayed behavioral response to threat (Messman-Moore & Brown, 2006), improving social support systems, and reducing alcohol and drug use and sexual risk-tasking (Edwards & Sessarego, 2018). Although there is no guarantee that women’s use of resistance strategies will be effective in the presence of someone willing to harm them, resistance interventions can increase women’s chance of effectively resisting or avoiding sexual victimization (Senn et al., 2015, 2017) and reduce self-blame no matter the outcome (Senn et al., in press).

A small number of studies have examined the use of resistance and self-defense tactics among women who have taken sexual assault risk reduction programs (including empowerment self-defense; ESD) that interrogate the social conditions enabling sexual assault and the psychological barriers to resistance. Participants who completed a risk reduction program (including 2.5 hours of self-defense) reported using assertive verbal tactics (61.5%), physical self-defense (6.5%), and yelling and running away (4.7%) in response to a perceived threat over the 6-month follow-up period (Gidycz et al., 2006). Of those women who used yelling and running away or physical self-defense, approximately 63% avoided being sexually assaulted. In two additional studies, Gidycz and colleagues found that, compared to the control group at the 4-month follow-up, risk reduction program participants were more likely to report attending to intuition, avoiding telegraphing emotions in an uncomfortable dating situation (Gidycz et al., 2015; Orchowski et al., 2008), using assertive body language (Orchowski et al., 2008), and yelling and running to escape an attacker (Gidycz et al., 2015). Gidycz et al. (2015) found that, compared to the control group at the 7-month follow-up, program participants were more likely to report attending to intuition, avoid telegraphing emotions, yelling and running to escape an attacker (as reported at the 4-month follow-up), and were more likely to use physical self-defense and assertive verbal responses when in a risky dating situation. These studies also demonstrated program effects for women’s increased use of self-protective strategies in dating situations with a new partner (e.g., providing their own transportation and meeting in a public place; Gidycz et al., 2006; Gidycz et al., 2015; Orchowski et al., 2008).

Hollander’s (2004) study of a small group of women following a longer and more intensive (45 hours) feminist ESD course provided further insight into women’s use of information and skills from risk reduction/resistance programs. In qualitative survey responses, women reported that the training had changed their daily practices to avoid dangerous situations, including implementing a range of precautionary strategies (e.g., locking doors) and having a heightened awareness (e.g., using one’s intuition). As Hollander noted, even more salient were the strategies that women implemented to manage potentially dangerous situations with strangers and acquaintances using forceful verbal resistance and other assertiveness strategies that maintain boundaries.

### The Current Study

Prior research on sexual assault resistance finds that women with and without a history of victimization and risk reduction/resistance education, engage in a range of strategies to avoid or prevent sexual assault, but the strategies used are unlikely to be effective and restrict women’s lives in significant ways. The important work of Gidycz et al. (2006, 2015) and Hollander (2004) begins to address the lack of scholarship on women’s use of resistance strategies following risk-reduction/resistance education (including ESD) and provides initial insight into the types and effectiveness of strategies women use. Hollander’s (2004) study begins to identify how ESD training, specifically, changes women’s everyday lives. However, a deeper understanding of the knowledge and skills beyond forceful physical and verbal resistance used by women who have taken resistance education is lacking. Furthermore, feminist scholars and sexual assault prevention experts have argued that feminist resistance interventions, including ESD, foster empowerment, do not blame women, and hold perpetrators responsible (e.g., Hollander, 2016; Radtke et al., 2020). How women take up these messages and integrate them into understandings of their own resistance is not yet known.

The current study offers a unique opportunity to examine women’s responses to sexual assault threat through their recalled, open-ended responses to real-life sexual assault threats following participation in an effective sexual assault resistance intervention called Enhanced Assess, Acknowledge, Act (EAAA; known to students as *Flip the Script with EAAA™*; for program development and evaluation, see Senn, 2011; Senn et al., 2011, 2015, 2017). We provide a brief overview of the program’s format, underlying theory, and efficacy to contextualize our analysis and discussion. The program is based on Rozee and Koss’s (2001) Assess Acknowledge Act (AAA) model of sexual assault resistance and is enhanced with emancipatory sexual education. It is delivered over 12 hours to small groups (< 20) of university women by highly-trained peer facilitators under the age of 30. Young women learn to identify their own sexual and relationship values and boundaries; to acknowledge risk for sexual assault, particularly from male acquaintances (without increasing fear); to identify and address their emotional and cognitive barriers to resistance; and to develop confidence and skills to verbally and physically fight back through ESD training based on Wen-Do Women’s Self Defence (wendo.ca).

Like other feminist risk reduction/resistance education programs (Senn et al., 2018), EAAA targets the beliefs, knowledge, and skills of individual women; assigns responsibility for sexual violence to perpetrators; and is built on the foundational understanding that sexual violence is embedded in a sociocultural context that enables it (Radtke et al., 2020). Feminist sexual assault resistance education aims to make women aware of this context, and in the case of EAAA, of their own sexual values and capacity to push back against assumptions about women’s sexuality, capabilities, and strength.

As a feminist program, EAAA frames sexual assault risk for women as present only when someone coercive is also present, critiques the construction and representation of women as either helpless victims or “superhuman” women immune to men’s violence, directly addresses woman-blaming, positions individual women as the only ones who know what is right for them in any given situation, and is future-focused, affirming that all survival is resistance and women with victimization histories did the best they could with the tools they had at the time. It supports women trusting themselves and their judgment and counteracts the idea that women should limit their freedom to keep themselves safe. While the program makes risk personally relevant to women in order to disrupt the optimism bias that impacts women’s ability to accurately detect risk, and the focus on individual application is necessary for the program to be effective, EAAA simultaneously counters sociocultural influences on sexual assault (Radtke et al., 2020; Senn et al., 2011).

There is strong empirical support for EAAA’s effectiveness. When evaluated in a randomized controlled trial (RCT), EAAA participation resulted in large reductions in attempted rape (63%) and completed rape (46%) and reductions in other forms of sexual victimization across 12 months. Most benefits are maintained for at least 2 years (Senn et al., 2015, 2017) with women’s rape myth beliefs and woman-blaming attitudes substantially reduced (Senn et al., 2017).

As the research team that assessed the efficacy of EAAA, we were interested in what content from the program was used by women after the program was finished. Notably, the program content was based on research that has identified how women respond to and resist sexual assault risk. Thus, we aimed to contribute to the broader literature on women’s responses to sexual assault threat and inform effective intervention development and policy. In short, the purpose of our study was to examine women’s use of the content learned in an effective sexual assault resistance education program.

## Method

### Participants and Data

Participants were 893 undergraduate women from three Canadian universities (see Senn et al., 2013, 2015, 2017, for methodology and outcome data). In the original randomized controlled trial (RCT), 451 women received EAAA. Data for the current article came from the quantitative and qualitative responses of the 445 women (98.7%) who took EAAA and completed at least one of the four follow-up surveys administered at 6-, 12-, 18-, and 24-months post-intervention. See Table 1 for demographic information. These women were asked the following questions at each follow-up:

1. How often in the past 6 months have you used techniques that you learned in EAAA to protect yourself? [Question (Q)1, open-ended, quantitative]
2. Which techniques did you use? [Q2, open-ended, qualitative text box]
3. Were they successful in repelling an attack? [Q3, closed-ended, quantitative]

**Table 1.** Demographic Characteristics of Participants (N = 445).

Characteristic	
Mean age in years (*SD*)	18.5 (1.2)
Racial identity	
White (%)	319 (72.8)
Black or African Canadian (%)	27 (6.2)
East Asian (%)	32 (7.3)
Other (e.g., South Asian and Middle Eastern) (%)	60 (13.7)
Heterosexual (%)	408 (91.7)
Living in university residence (%)	240 (53.9)
Sexually active (%)	278 (62.5)
Currently in a romantic relationship (%)	204 (45.8)
Currently in a sexual relationship (%)	201 (45.2)
Ever dated a male (%)	381 (85.6)
Previous sexual assault training (%)	17 (3.8)
Previous self-defense training (%)	158 (35.5)
Sexual assault experienced since age 14 years	
Rape (%)	101 (22.7)
Attempted rape (%)	112 (25.2)
Coercion (%)	95 (21.3)
Attempted coercion (%)	122 (27.4)
Unwanted sexual contact (%)	208 (46.7)

### Data Analysis

We analyzed the data using descriptive statistics (Q1 and Q3) and content and thematic analysis (Q2). One hundred and ninety women indicated they had used techniques/strategies learned in the program in Q1, but five of these participants did not provide a response to the open-ended qualitative question. Data for the qualitative analysis (Q2) came from 372 qualitative responses across the four follow-up time points provided by 185 participants. The average length of qualitative responses was 19 words (range = 1–126, median = 15).

We conducted two separate but related analyses of women’s qualitative responses. The first was a content analysis using a theory-driven approach informed by program theory and content. This analysis identified the types and frequencies of EAAA strategies that the women reported using in their lives. The second was an inductive, data-driven thematic analysis examining the contexts in which women’s resistance took place to better understand what women’s resistance looked like in their everyday lives and determine what this can tell us about feminist sexual assault resistance broadly.

### Content Analysis

Our analytical framework for coding, analyzing, and interpreting women’s resistance strategies was similar to the dual approach used by Masters et al. (2006). Content codes were generated both inductively and deductively and responses were often coded under multiple codes. The development of inductive codes was informed by the program theory and content (Nurius & Norris, 1996; Rozee & Koss, 2001; Ullman, 1997). We began with an initial set of codes based on Ullman’s (1997) categories of effective resistance (e.g., forceful physical, non-forceful verbal, and leaving) that aligned with the self-defense knowledge and techniques taught in EAAA. Women’s responses were coded as (a) forceful verbal resistance, operationalized as active verbal strategies that are said forcefully and with urgency aimed at stopping the attack, scaring the offender and/or attracting outside help; (b) non-forceful verbal resistance, operationalized as non-aggressive verbal responses to attack, such as pleading; (c) forceful physical resistance, operationalized as active, aggressive behaviors enacted by the victim directly against the offender to stop an attack; (d) non-forceful physical resistance, operationalized as passive, physical resistance techniques used by the victim to evade the offender’s attack; and (e) leaving. Responses coded under one or more of these five categories indicated resistance to an imminent attack (i.e., a specific and immediate sexual assault threat that likely would have resulted in a completed sexual assault).

If a participant response did not fit into an existing code, a new code was co-created by the research team through a collaborative and iterative process. The data were initially coded by a trained research assistant and reviewed and revised by the first, second, and fourth authors until there was 100% agreement on the coding of each response. The final coding scheme had 19 codes (see Table 2 for the deductive codes that go beyond Ullman’s categories). Fifty-six (77.8%) responses were coded under both Ullman’s resistance categories and the deductive codes, because the women tried to preempt the assault and then had to deal with it more directly (presumably because the threat escalated).

**Table 2.** Women’s Strategy Use to Preempt the Progression of Aggressive/Coercive Behavior Beyond Ullman’s (2007) Resistance Strategies.

Knowledge/Strategy	*n*	%
Use of assessment strategies (e.g., being aware of environment; avoiding men who display danger cues)	88	47.6
Leaving a situation preemptively	83	44.9
Building alliances	51	27.6
General assertive communication (e.g., “saying no”)	52	27.6
Action prompted by listening to gut/intuition	38	20.5
Assertive communication about unwanted sex	38	20.5
Taking precautions (e.g., planning a meet-up spot with friends in the event they are separated at the bar)	23	12.4
Reducing risk related to unfair gender roles expectations (e.g., providing own transportation)	17	9.2
Assertive communication about wanted sex	10	5.4
Body assertiveness (e.g., walking with confidence)	7	3.8
Awareness of sexual rights	3	1.6
Nonverbal body assertiveness (e.g., “standing my ground”)	2	1.1

*Note.* Total frequencies were calculated over four follow-up time points at 6, 12, 18, and 24 months following the completion of EAAA.

To facilitate the organization and analysis of the content codes, data were coded in Excel and SPSS. Composite variables were created in SPSS to allow for descriptive quantitative analysis. Missing data were excluded from the analysis.

### Thematic Analysis

While the content analysis allowed for the documentation and quantification of the full range of EAAA knowledge and skills that women reported using, the thematic analysis examined the social context surrounding women’s accounts of their resistance and documented how women understood their acts of resistance. Specifically, we were interested in how women framed their use of EAAA strategies and how this framing related to broader sociocultural discourses and assumptions about women’s resistance. Our approach, informed by Braun and Clarke’s (2019) reflexive thematic analysis, used a realist epistemological orientation to examine the knowledge and skills women reported using. Further, our interpretation of women’s responses was informed by our positionality as violence against women researchers, the philosophy and content of the program (e.g., the feminist messaging that women are not responsible for sexual violence and have a right to defend their boundaries), our background knowledge of non-feminist anti-rape programs and campaigns, as well as the extant literature on women’s behavioral responses to sexual assault risk/use of protective strategies.

Data analysis was guided by the recursive phases of reflexive thematic analysis (Braun & Clarke, 2006, 2012, 2019). Individual responses were initially coded (labeled) by the first author for latent (i.e., explicit) and semantic (i.e., underlying) meanings, and codes were discussed and refined by the first and third author. This collaborative team-based approach was intended to produce “a rich and more nuanced reading of the data rather than seeking consensus on meaning” (Braun & Clarke, 2019, p. 594). As the question to which the women responded (“what techniques did you use?”) was not designed to elicit in-depth narratives, the written responses were often quite brief and were often labeled with only one code. Consistent with Braun and Clarke (2019), we understand themes to represent “patterns of shared meaning underpinned or united by a core concept” (p. 593).

## Results

### Frequency of Strategy Use and Success

In response to Q1 (“How often in the past 6 months have you used techniques that you learned in EAAA to protect yourself?”), 42.7% of women (*n* = 190) reported using EAAA strategies at least once in the following 2 years. Among these women, the frequency of reported use ranged from 1 to 100, with an average of almost 6 times (*M* = 5.89*, SD* = 10.01, *Mdn* = 3.00). Thirty-nine percent of these women (*n* = 74) indicated they had used EAAA strategies once or twice, 30% (*n* = 58) had used them 3 to 5 times, 18% (*n* = 34) 6 to 10 times, and 11.5% (*n* = 22) more than 10 times. A higher proportion of survivors (60.7%) than other women (35.7%) used strategies they learned in the program, χ^2^(1) = 24.08, *p* < .001. There were no other demographic differences between women who used strategies and those who did not. Sexual assault survivors (reported attempted and/or completed rape at baseline) also used significantly more strategies (*M* = 3.99) than other women (*M* = 2.07), *F*(1, 432) = 5.79, *p* = .02.

Frequency analyses were conducted to determine the number and percentage of women who indicated that their efforts to resist sexual assault using what they learned in EAAA were successful (Q3). Of the 190 women who reported using EAAA strategies to protect themselves, 149 (78.4%) reported that their efforts were successful, 30 (15.8%) reported their efforts were both successful and unsuccessful (i.e., they reported using strategies at multiple follow-up time points and indicated some efforts were successful while others were not), and only seven women (3.7%) said their efforts were unsuccessful. Responses are missing for four women, of whom three also did not provide an open-ended qualitative response.

### Content Analysis

#### Strategies Used in Response to an Imminent Threat

Of the 74 women who reported using strategies in response to an imminent threat at least once, 31.1% (*n* = 23) reported using more than one strategy across 2 years. The most common response to imminent sexual assault threat was forceful physical resistance: 42 women (22.7%) reported actions such as pushing off, hitting (e.g., using hand strikes against the perpetrator), elbowing, and stomping on the perpetrator’s foot at least once. For example, one participant described “slamming the base of the hand into the bottom of his nose.” The second most common response, non-forceful verbal resistance (e.g., making up an excuse to leave) was employed by 29 women (15.7%) at least once. For example, one participant reported that she “called a friend to come to a room and pretend they needed me to come with them.” Fourteen women (7.6%) reported using forceful verbal resistance such as yelling or loudly drawing other people’s attention. For example, one participant reported that “In broad daylight, a random man had come up to me when I was waiting for the bus and tried to get me to have sex with him. He got in my personal space and touched my thigh. I kept telling and yelling at him to leave me alone.” A smaller number of women reported leaving the situation (*n* = 12, 6.5%) in response to a direct threat. For example, one participant reported that she used multiple tactics including leaving saying, “used screaming, physical force - kicked him off, called my mother, ran out of the house.” The least common strategy in response to a sexual assault threat was the use of non-forceful physical resistance. Only five women (2.7%) reported using non-forceful physical resistance such as removing a man’s hands or moving their legs away from him. For example, one participant reported “pulling away from someone who cornered me.”

#### Strategies Used to Preempt the Progression of Aggressive/Coercive Behavior

Although Ullman’s (1997) categories of resistance were useful in capturing the meaning of the women’s responses to an imminent threat or attack, those responses aimed at preempting men’s coercion and assault (e.g., risk assessment and trusting one’s “gut”) were not codable under this framework. This latter group of responses were of interest because they are part of the knowledge and skills included in EAAA. In this way, participant responses showed a broader understanding of the question, “what techniques did you use to protect yourself” than was anticipated based on Ullman’s (1997) previous research. Women reported using a diverse range of strategies to interrupt the progression of aggressive or coercive behavior (see Table 2). Notably, almost half of the women who took EAAA (47.6%, *n* = 88) employed assessment strategies to reduce their sexual assault risk, including both situational assessment (i.e., being aware of surroundings, avoiding isolation, and reducing/being aware of alcohol risk) and behavioral assessment (i.e., recognizing and avoiding men who display risk cues). For example, one participant reported the following situational assessment: “I have been able to spot signs better that could lead to a possible uncomfortable situation. I also always make sure I can get out of where I am (knowing where the exit is and where I am).” Another participant reported using the “techniques/red flags to recognize a potentially dangerous man.” Leaving the situation preemptively was the second most commonly used strategy (44.9%, *n* = 83). This strategy is an extension of leaving in response to a specific threat but occurs earlier on when sexual assault risk has been acknowledged but has not yet escalated. For example, one participant described how she left the situation before it became dangerous:

I used a buddy system when I went to a bar and was meeting a boy, and so when we found out he was kind of creepy we got a bouncer to talk to him then my friends and I left. Instead of telling that boy where I lived so that he could go to the bar with us, I had said to just meet us there, which ensured he was not able to follow us back.

Other commonly used strategies included building alliances with other people, especially women friends (27.6%, *n* = 51; e.g., “I try to bring my friends if I go to a party”) and general assertive communication (e.g., saying “no”; 27.6%, *n* = 52) that women perceived as having protected them in a specific situation(s).

### Thematic Analysis

This analysis developed four themes that captured the specific ways that the women protected themselves and other women while acting in opposition to oppressive social norms: (a) appraising situations through a gendered lens, (b) naming discomfort and taking action to increase comfort and safety, (c) explicitly subverting gender norms, and (d) resistance as a community act.

#### Appraising Situations Through a Gendered Lens (Theme 1)

Whereas the content analysis identified assessment (of situations, men’s behavior) as a common strategy to preempt sexual assault risk, the thematic analysis showed that such assessment involved applying a gendered analysis in social situations with men. This entailed an acknowledgment of the unfortunate reality that men’s actions may be malicious and can be anticipated when sexual assault risk cues are present in their behavior (e.g., persistence, power, and control) or the social situation (e.g., presence of alcohol and isolation). That is, women recognized that any man may become sexually coercive within a patriarchal society that entitles them to be sexually dominant, but through using resistance tactics strategically to undermine this reality in specific situations she could continue living her life with relative freedom.

Women’s accounts indicated an awareness of the different ways that men exert power and influence over women in intimate and social situations. For example, one woman reported recognizing a large number of men in her vicinity as potentially unsafe:

We were at a party and on the dance floor when I realized the large ratio of men to women at the party, and I didn’t feel safe and I told my friends and we moved off the dance floor for a while.

She implicitly acknowledged the gender imbalance in this particular situation, which could be intentional or strategic and constitute a power move by the men. Other women made explicit their judgment that men’s behavior was an intentional attempt to facilitate sexual activity by rendering them less able to resist: “I don't allow guys to buy me drinks at the bar if I know or suspect they are only trying to get me drunk.” Another woman reported that “[I]… kept my feet planted on the floor while watching a movie with a male acquaintance alone. I was able to stand up when he got too frisky.” This woman anticipated how the situation (watching a movie with a male acquaintance) could unfold (him getting “frisky”) and in not wanting such attention was prepared to resist if this came to pass. She recognized that isolation with a *male acquaintance* increased her risk for coercion and enacted a strategy to reduce this risk without limiting her ability to socialize and enjoy his company (up until the point where he acted in a way that was counter to what she wanted and was potentially unsafe). These accounts show that in learning how to assess situations for sexual assault risk, the women also came to understand that such situations are risky by virtue of some men’s willingness to exert power in order to sexually coerce women, an exercise of power that is not generally available to women.

#### Naming Discomfort and Taking Action to Increase Comfort and Safety (Theme 2)

Women often named their discomfort when their asserted boundaries were ignored, thereby prioritizing their well-being in their actions. For example, one woman said:

Verbal communication. I was seeing a guy and he wanted to go further than I did. He was more forceful than I felt comfortable, and he wasn’t listening. I used “NO,” “get off of me,” “I’m not having sex,” “Stop,” in a loud, firm voice. I felt powerful and confident to stand up for myself, and he backed off.

Recognizing that she was uncomfortable in the situation, she responded with forceful verbal resistance that continued until she was safe. Importantly, this quote is representative of the data set as a whole in which women positioned men’s behavior and persistence—not what the women themselves did or did not do—as problematic.

In addition, most women positioned themselves as agentic in their resistance:

A guy I didn't feel comfortable or safe around used to give me rides to school (which is about an hour drive from my home) after taking the program I learned that if my gut’s telling me there is something wrong then I need to trust myself and I also learned that lying is sometimes ok to get out of bad situations. So I told him I won’t be needing his car rides because my family would drop me at school, and from that day on I started taking the bus and I feel more confident in myself now.

As reflected in this account, women made judgments about whether they were comfortable in a particular situation and chose a course of action that allowed them to actively reclaim their comfort and safety in a way that prioritized their own needs and well-being. The exploring/setting of boundaries and the “action” taken after those boundaries have been crossed are central elements of EAAA and, thus, this theme reflects the knowledge and skills taught within EAAA. Attending to “what feels good to me” constituted a form of self-trust that served to foster personal well-being.

#### Explicitly Subverting Gender Norms (Theme 3)

Women’s verbal or physical assertion in response to men’s aggression demonstrates a rejection of the socialization that encourages women to remain quiet and polite for the sake of men’s feelings and egos. The content analysis indicated that approximately half of the sample reported being verbally and physically assertive in the face of an attack. The thematic analysis indicated that assertive responses were one way in which the women subverted gender norms. For example, one woman reported that “I was able to stop the situation in its tracks by speaking assertively to the guy telling him to stop,” and another woman said:

Using my voice to tell the man that I didn't want his sexual advances, has been a very commonly used technique and works like a charm if used immediately. It usually comprises of me specifically and sternly saying, “Don't touch me.”

Unlike the accounts associated with Theme 2, the women’s accounts associated with Theme 3 do not acknowledge a sense of discomfort or other internal psychological state. Rather, they are focused on the direct and assertive action (e.g., being upfront about why they are leaving rather than making an excuse) women took in response to men’s assumptions, boundary-crossing, and persistence. As another example, some women reported refusing to acquiesce or defer to men’s preferences or desires in a given situation:

When a man wanted to take me out on a date and then asked that if instead of going out, we just hang out at his house. I told him that I would not come over to his house and discontinued communication with him.

The last-minute change in plans from being in a public place (going out on a date) to potentially being alone in an isolated situation (hanging out at his house) increased the risk to her. In refusing his suggestion and cutting off communication, she prioritized her needs and safety over his feelings. Further, she violated the heterosexual dating script that gives the man responsibility for organizing the event, and she was explicit in her reasoning for not coming over.

Other examples of the subversion of gender norms and expectations included, “Paying for my own dinner when a man was extremely forceful in doing so” and “I was mean after he didn't get the idea of ‘No.’ I embarrassed him in front of lots of people.” These explicit accounts of subversion that were beyond being vocally/physically assertive offer striking examples of how women actively engaged in resisting gender norms and expectations, such as a man paying for dinner on a date or women being mindful of men’s feelings so as to not embarrass them.

#### Resistance as a Community Act (Theme 4)

The content analysis identified building alliances with other women/friends as a commonly used resistance strategy. Through thematic analysis, we examined the social context in which these alliances unfolded, leading us to see that the women were actively and meaningfully involved in resistance together. For example, one woman spoke about online dating:

I had a date with a guy that I met on Tinder, so I made sure to meet him in a public place and I had one of my friends come with me to ensure that I would not be in danger of sexual assault.

In addition to avoiding isolation by meeting in a public place, she further reduced her isolation by partnering with her friend to ensure her safety. Notably, she was not restricting her behavior here; her resistance strategy was not to stay at home, stop using Tinder, or avoid men altogether. Rather, she controlled the situation to prioritize her own safety and feelings of comfort.

In a different example of how women’s communities, often friends, are actively involved in their resistance to men’s sexual aggression, one woman shared how she and her friends take care of each other when out dancing:

At the bars or clubs when a guy is getting to handsy or I no longer want to dance with him and he won’t let me go then my friends and I always make sure we are around each other so when we shake our heads we know to help each other get out of the situation.

This woman and her friends had developed an enhanced buddy system that allowed them to enjoy themselves dancing at the bar with men but put in place a collective exit strategy when their boundaries had been crossed or they were no longer safe. Notably, this woman’s account also speaks to the frequency with which resistance strategies need to be implemented by women to maintain their safety. Other women spoke about “watching out” and “keeping an eye” on each other: “I always make sure my friends are within sight when we go out and we never leave without advising each other,” and about sharing EAAA information, including the self-defense strategies, with other women. As these examples illustrate, women actively created partnerships with other women both to preempt men’s coercive behavior and to interrupt that behavior in the moment and demonstrated care for each other’s safety and well-being. Building alliances with other women to achieve a common goal is clearly consistent with feminist practice.

## Discussion

In this study, we examined women’s resistance to sexual coercion and assault following participation in a feminist sexual assault resistance intervention. Our open-ended approach to data analysis allowed for self-defense and resistance strategies other than those already identified in the literature. As part of the RCT follow-up surveys (the source of the data for the current study), women were asked about their use of program content, but we remained open to the possibility that what the women identified as strategies used to protect themselves and what we as researchers identified as risk reduction/resistance based on the literature might differ. In addition to counting reported strategies, we used thematic analysis to examine more closely women’s understandings of their resistance, paying particular attention to how this related to program content and the broader social context.

Forty-three percent of the women reported using EAAA strategies to protect themselves in the 2 years following their participation. Women reported using a myriad of resistance strategies in response to imminent and potential sexual assault threats and employed those strategies most likely to facilitate the successful interruption or avoidance of sexual assault (i.e., forceful physical and verbal strategies, leaving; Ullman, 1997, 2007). Of the five main types of resistance on which much of the previous research on women’s sexual assault resistance has focused (i.e., Ullman’s [1997] resistance categories), forceful physical resistance was the most commonly used. Forceful verbal resistance was less common, suggesting women may have felt confident and entitled to use forceful physical resistance as their first line of defence against an imminent threat. The less frequent use of forceful verbal resistance could also be explained by the types of situations in which resistance was needed. Women’s use of resistance often took place in bars, which tend to be noisy, thereby making verbal communication difficult. Furthermore, women’s use of a range of strategies to preempt coercive behavior may have resulted in fewer women needing to employ forceful physical and verbal strategies, because they were able to identify and respond to sexual assault risk at an earlier stage (e.g., by leaving or using assertive communication) in a way that undermined men’s ability to escalate to a more imminent threat. This interpretation is supported by the large reduction in attempted rape shown in the program outcome evaluation (Senn et al., 2015).

Most women (78.4%) who employed at least one EAAA strategy indicated their efforts were entirely successful in repelling or avoiding an assault. Only 3.7% of women indicated their efforts were entirely unsuccessful. This is consistent with the outcome evaluation that showed significant reductions in completed rape, attempted rape, and forced sexual contact (in addition to other types of sexual victimization) among program participants (Senn et al., 2015, 2017). It is important to note that 45% of the women providing these responses and evaluating the success of their resistance were survivors, providing further evidence of the generalization of program benefits for women with and without a history of sexual assault (Senn et al., 2015, 2021).

Examining women’s accounts of the information and skills from EAAA they used not only identified which components of the program women integrated into their lives, but enhanced understanding of the mechanisms that made EAAA effective in reducing sexual victimization, rape myth acceptance and victim-blaming attitudes, and increasing self-defense self-efficacy and risk perception. Mediation analyses of EAAA showed that the program’s positive effects on situational risk perception and willingness to use forceful resistance (measured using a hypothetical scenario) combined with improvements in self-defense self-efficacy explain the reductions in attempted and completed rape following program participation (Senn et al., 2021). The current study complements and extends these findings by documenting how women resisted subsequent sexual assault threat(s) and the types of situations that required women’s resistance (e.g., being approached at a bus stop, hanging out with a date, and dancing at the bar).

Despite their potential for promoting the health and well-being of girls and women by reducing sexual victimization, resistance interventions that teach women how to undermine risk and effectively resist coercion have been criticized by some feminists as another way to restrict or blame women. This criticism is, in part, rooted in problematic awareness campaigns with “advice” for girls and women on how to stay safe (e.g., anti-drinking posters, Weiss, 2017) and previous research documenting the extent to which women modify and restrict their lives to protect themselves from sexual assault (Gordon & Riger, 1989; Stanko, 1987), for example, not having relationships with men and not leaving the house (Ullman et al., 2018). The majority of women’s first-hand accounts of resistance in the current study do not lend support to this criticism. Beyond the specific strategies that women employed, the themes identified in women’s accounts of resistance suggest that resistance following participation in EAAA is a form of feminist practice. That is to say, women prioritized their well-being while pushing back against sociocultural norms when responding to an actual or perceived sexual assault threat, or to a man who they perceived as entitled. Their acts of resistance served to keep them and other women safe in the moment, while also challenging or disrupting patriarchal and other social scripts.

Importantly, as demonstrated by the thematic analysis, these acts of resistance did not require placing restrictions on their lives. Rather than preventing women from doing what they wanted to, their use of resistance strategies from EAAA may have enabled them to do what they wanted (e.g., going to the bar rather than staying home, or dating someone they met through a dating app). In contrast to constructions of women as perpetual victims who must limit their exposure to risk in order to stay safe, women’s accounts were almost entirely absent of restrictive behavior such as staying at home, avoiding men in social or dating situations, or engaging in precautionary or protective behaviors that upheld damaging rape myths, such as not wearing certain clothes to avoid being seen as inviting attention. Rather, women’s accounts referenced behaviors and activities that are often actively discouraged in non-feminist campaigns and risk reduction education targeting women, such as drinking alcohol, being in bars/clubs, dating casually or non-exclusively, and engaging in casual sexual activity.

While we cannot definitively say women’s approaches to the situations they encountered were caused by the program, because these qualitative data were gathered only from the group of women who took the program and not from those who were randomly assigned to the control group, our claims are supported by the quantitative evidence from the RCT which does support causal conclusions. For example, we have reported elsewhere that women who took EAAA experienced substantial reductions in general and specific (woman-blame) rape myth beliefs compared to the control group (Senn et al., 2017) as well as lower self-blame if they experienced rape post-program (Senn et al., in press). As such, our findings provide initial evidence that women received the program’s empowerment message that they can trust their judgment and do not need to limit their freedom to increase their safety. Women’s responses reflected a sense of agency and empowerment in decision-making when responding to perceived or real threats of sexual coercion and assault. For example, participants reported having increased feelings of confidence and comfort as a result of the actions they took to prioritize their own needs and safety. Women consistently positioned themselves as actors who had control over the situation (e.g., taking control of the plans for going on a date, including the location and who was present) and chose a course of action that was in their best interests, including leaving the situation or ending the relationship. Importantly, women did not construct preventative resistance measures (e.g., leaving the dance floor) as their responsibility and often explicitly placed the responsibility for the risky situation on men (e.g., “he wasn’t listening to me”). Women’s resistance to this “critical element” of rape culture (i.e., woman-blaming; Radtke et al., 2020) suggests that the criticism of risk reduction/resistance programs as inherently woman-blaming does not apply to those programs that are explicitly feminist in philosophy and practice.

Criticisms of women’s self-defense training specifically, which is a key component of feminist sexual assault resistance education, include the belief that it is ineffective, focused on stranger rape, encourages victim blaming, and fails to target the root causes of sexual violence (reviewed by Hollander, 2016). Women’s accounts of their resistance, including use of forceful physical and verbal self-defense, push back against these criticisms and align with Hollander’s (2016) assertion that ESD training “interrogate[s] both the social conditions that facilitate sexual assault and the psychological barriers to women’s resistance that result from gender socialization and expectations” (p. 209). Feminist sexual assault resistance programs that include ESD such as EAAA may offer women an alternative way to think about and manage sexual assault risk. In the current study, women reported using information and skills from the program in ways that disrupted gender norms and sexual scripts (e.g., being assertive in boundary setting in sexual and non-sexual situations), counteracted heterosexual dating scripts (e.g., refusing drinks or meals from men), and reclaimed space for women. This is consistent with Hollander’s (2004) findings that taking ESD changed women’s everyday lives. These findings further suggest that EAAA’s approach—which frames men’s violence not as the actions of deviant or “sick” men but rather “everyday” men emboldened by a sociocultural context supportive of men’s violence against women—may allow women to acknowledge and effectively undermine personal risk in a way that offers them some degree of agency and empowerment, while viewing sexual violence within this broader sociocultural frame.

We acknowledge the limits of understanding “choice” within a neoliberal context and broader violence prevention and crime discourses that make risk management of men’s violence part of the subjectivity of women’s lives and how this violence functions as a form of self-policing for women (e.g., Stanko, 1997). At the same time, we believe that women deserve access to information and skills that may help them not only effectively resist sexual assault but engender social change. Further, feminist sexual assault resistance programs like EAAA may play a role in disrupting the traditional violence prevention discourse that constructs women as simultaneously empowered and victimized (Frazier & Falmagne, 2014). How women in the current study resisted and subverted gendered social norms and socialization in their resistance to men’s coercion is part of broader sociocultural and historical shifts in gender and social norms that are, albeit slowly and non-linearly, challenging the acceptability of violence against women (see Radtke et al., 2020). Thus, findings from the current study provide support for the position that feminist sexual assault resistance education (including ESD) may function as a form of primary prevention (Hollander, 2016; McCaughey & Cermele, 2015). Despite the potential of resistance education, we are firm in our belief that perpetrators are responsible for the violence they commit and thus responsible for ending sexual violence and that women and others at high(er) risk of sexual assault should not bear the responsibility of engaging in self-protective behaviors. There remains a pressing need for effective interventions targeting perpetration.

### Practice Implications

The primary application of this study’s findings is in the development of future sexual assault resistance interventions (Anderson et al., 2016) and the refinement of EAAA. For example, knowing what knowledge and skills women remember from EAAA and successfully employ up to 2 years later can inform the strengthening of the program (e.g., can the program do more to reduce self-consciousness and encourage forceful verbal resistance if emotional obstacles to the use of this effective strategy remain?) and the development of booster sessions. Furthermore, the findings support the efficacy of the program’s theory and curriculum (information and exercises) in engaging participants and facilitating deep learning.

Moreover, this study expands the focus of the existing literature on women’s sexual assault resistance from a narrow concern with women’s behavioral responses to sexual assault threat to include the breadth of knowledge (e.g., “I learned that lying is sometimes ok to get out of bad situations”), messaging (e.g., trusting your intuition), and behaviors (actions) acquired through an effective resistance intervention that women integrated into their daily lives. This study contributes to our understanding of how women respond to sexual assault threat following resistance education and demonstrates how one particular feminist program leads to the use of protective behavioral strategies in acquaintance situations (instead of the unlikely stranger situation) and do not require women to socially isolate or take responsibility for men’s sexual objectification of them. These additional aspects of women’s resistance offer a more fulsome understanding of women’s resistance that begins to account for the cognitive, emotional, and social aspects of women’s resistance made visible in Rozee and Koss’s (2001) outline of the AAA model.

Finally, it is worth noting that access to EAAA and other prevention programs are currently largely restricted to post-secondary institutional settings, which, at least in North America, are predominantly White and middle-class. EAAA is being adapted for other contexts and populations, including trans and gender diverse students and adolescent girls, but differential access to effective programming based on life circumstances persists. There is much work to be done outside of university and college campuses to increase the accessibility of effective sexual assault prevention programming.

### Limitations and Future Research

The most significant limitation of the study is the potential for recall bias. Participants were asked to recall what information and skills they used from the program in the previous 6 months (for 2 years), as well as the number of times they had used this information or skill. This recall was likely more accurate for participants who used fewer strategies on fewer occasions. However, several participants offered detailed accounts of their experiences suggesting that recall was not generally, negatively affected. It is also possible that program knowledge use could be underestimated as women may later use program content without remembering where they acquired it. This was to be expected given that scripts for delivering the program direct facilitators to purposefully draw on and expand women’s own knowledge to increase their trust in themselves. In future research, using a prospective design, such as daily diaries, could be beneficial in mitigating memory biases and promote consistency in the detail provided in women’s accounts.

While the sample in the current study represents the diversity of the three universities from which they were drawn, it is a predominantly White, heterosexual sample. This is a limitation for exploring the knowledge and skills used by other specific subgroups of women. However, the efficacy of EAAA has been demonstrated for women of varying sexual and racial identities (Senn et al., 2019). Further, many of the strategies women reported using were not employed in a specifically heterosexual romantic or sexual context. Nevertheless, future research should attend to what works for women occupying varied social positions.

Length of the accounts varied greatly in our study, with brief responses typically providing less and sometimes no context about the woman’s resistance and/or the situation that prompted the resistance. To encourage participants to provide sufficiently detailed responses, future retrospective and prospective research on women’s sexual assault resistance may benefit from providing prompts to be as detailed as possible and consider particular pieces of information (e.g., location and relationship to the perpetrator) relevant to the research question(s). Relatedly, while we expected that women’s responses would reflect situations where they faced an imminent threat and that their reported resistance strategies would align with what is typically reported in previous research (e.g., use of forceful physical and verbal strategies), their responses offered a broader and more comprehensive look at women’s resistance following sexual assault resistance education. While this is a strength of the current study and extends existing research on women’s resistance beyond the traditional categories of resistance, future research would benefit from asking women to specifically indicate if their resistance was in response to their perception of an imminent threat. This would provide further insight into the types of resistance strategies women use in response to different contexts for sexual assault threat.

### Conclusion

In this study, we examined women’s use of resistance strategies to respond to the threat of sexual coercion and assault following participation in EAAA. Our findings are consistent with previous research indicating that women use a wide variety of strategies to resist sexual coercion, both in response to the imminent threat and as part of a broader attempt to preemptively interrupt or avoid men’s sexual coercion. Furthermore, we identified the knowledge and skill components of EAAA that women said they learned and were able to successfully employ in response to sexual assault threat up to 2 years after completion of the program. Counter to criticisms that risk reduction/resistance programs blame women or make them responsible for stopping men’s violence, women in the current study typically positioned themselves as agentic and empowered in their resistance. Furthermore, women’s acts of resistance served to keep themselves and other women safe in the moment while also challenging or disrupting patriarchal and other social scripts and norms.

These findings have practical application for the adaptation of EAAA for other populations and contexts and the development of additional sexual assault resistance interventions or booster sessions. Feminist sexual assault resistance interventions that effectively reduce victimization should be made available to young women on university and college campuses (and beyond) as part of comprehensive sexual assault prevention frameworks (Orchowski et al., 2018).
